# Subtalar arthroereisis combined with medial soft tissue reconstruction in treating pediatric flexible flatfoot with accessory navicular

**DOI:** 10.1186/s13018-023-03542-w

**Published:** 2023-01-19

**Authors:** Chao Shi, Mingxiu Li, Qiu Zeng, Xiaodong Wen, Feng Tian, Yi Li

**Affiliations:** 1 Department of Foot and Ankle Surgery, Honghui Hospital Affiliated to Medical College of Xi’an Jiaotong University, Xi’an, 710054 Shaanxi Province China; 2 The Second Clinical Medical College of Shaanxi University of Chinese Medicine, Xianyang, 712046 Shaanxi Province China

**Keywords:** Flatfoot, Arthroerisis, Accessory navicular, Posterior tibial tendon

## Abstract

**Purpose:**

Accessory navicular is accompanied by the deformity of valgus flexible flatfoot. The surgical treatment includes reconstruction of insertion of posterior tibial tendon following resection of the accessory navicular. However, this treatment could not correct completely the deformity of valgus flexible flatfoot. This study aimed to evaluate the efficacy of subtalar arthroereisis combined with medial soft tissue reconstruction in treating 8–14-year-old flexible flatfoot patients with accessory navicular.

**Methods:**

Clinical data of 35 pediatric flatfoot patients (with 50 feet) with accessory navicular who underwent subtalar arthroereisis and medial soft tissue reconstruction between April 2013 and September 2018 were analyzed retrospectively. Anteroposterior, lateral, and hindfoot alignment radiological images were measured in the weight-bearing position, and visual analog scale (VAS), American Orthopedic Foot and Ankle Society (AOFAS) ankle-hindfoot scale, and satisfaction degree were evaluated. Also, surgical complications were recorded.

**Results:**

The average follow-up time of the patients was 30 ± 9.3 months. None of the patients presented wound complications, and no implant loosening was detected. The AOFAS and VAS scores improved significantly (*P* < 0.001). Radiological parameters, such as the talar first metatarsal angle and talonavicular coverage angle on anteroposterior foot view, Meary’s angle and calcaneal pitch angle on the lateral view, and calcaneus valgus angle on hindfoot alignment view improved significantly (*P* < 0.001). Postoperative complications were observed in three patients.

**Conclusion:**

Subtalar arthroereisis combined with medial soft tissue reconstruction significantly alleviated pain and improved the functions in pediatric and adolescent flexible flatfoot patients with accessory navicular; also, the radiological manifestations and functions improved.

## Introduction

Flatfoot with accessory navicular refers to flatfoot caused by posterior tibial tendon (PTT) dysfunction induced by accessory navicular. The major clinical manifestations include painful medial navicular tuberosity and collapse of the medial longitudinal arch, accompanied by different degrees of forefoot abduction and hindfoot valgus deformity [1].

PTT is the largest and most anterior of the medial ankle canal and the primary dynamic stabilizer of the medial longitudinal arch [2]. PTT dysfunction induces the collapse of the medial longitudinal arch and gradual reduction in tendon strength, thereby resulting in various typical secondary deformities of flatfoot [3]. Accessory navicular alters the traveling and insertion of PTT and attenuates the function of PTT in arch lifting and foot introversion, thereby damaging the arch biomechanics and leading to flatfoot [4]. Recent studies have shown that accessory navicular is associated with flatfoot, which induces or worsens the accessory navicular-related symptoms. Simply treating painful accessory navicular could not correct the flatfoot deformity satisfactorily [1, 5–7]. The progression of flatfoot deformity induces the progressive collapse of the arch, leading to rigid flatfoot and arthritis [8, 9].

In pediatric and adolescent patients with flatfoot, approximately 12% exhibit accessory navicular [4]. The surgical treatments for pediatric flatfoot patients with painful accessory navicular include two parts as follows: (1) treat the symptomatic accessory navicular and (2) correct the flatfoot deformity and related symptoms. Kidner procedure reconstructs can alleviate or eliminate the pain in navicular tuberosity. However, whether could persistently correct the collapse of the arch is yet to be elucidated [7]. Subtalar arthroereisis is an effective method for the treatment of flexible flatfoot in children [10–12]. To date, we have applied osteotomies combined with soft tissue procedures to treat symptomatic flexible flatfoot deformity in children [13]. However, no studies have yet investigated the short- and moderate-term efficacy of subtalar arthroereisis combined with medial soft tissue reconstruction in treating Chinese pediatric flatfoot patients with accessory navicular. Thus, the present study aimed to describe the radiological and clinical outcomes using subtalar arthroereisis combined with medial soft tissue reconstruction in pediatric flatfoot deformity with accessory navicular.

## Materials and methods

### General characteristics

Data of 36 flexible flatfoot patients with accessory navicular underwent subtalar arthroereisis combined with medial soft tissue reconstruction in the Xi’an Jiao Tong University affiliated Honghui Hospital between April 2013 and September 2018 were analyzed retrospectively.

The inclusion criteria were as follows: (1) Flexible flatfoot patients with accessory navicular: with medial midfoot protuberance and touching pain, flatfoot in weight-bearing view, accompanied by forefoot abduction and hindfoot valgus, and relevant clinical manifestations (pain at the protuberance of accessary navicular or fatigue or pain in the lower limbs after long-time walking or physical exercise). X-ray imaging also confirmed the presence of accessary navicular accompanied by flatfoot deformity; (2) age 8–14-years-old; (3) after ≥ 6 months of conservative therapy, the effects were suboptimal, deformity or symptoms worsened progressively.

The exclusion criteria were as follows: (1) neuromuscular disease or tarsal coalition; (2) history of bone fracture of the affected foot; (3) evident articular degeneration; (4) incomplete data. This retrospective study was approved by the Institutional Review Board. All the patients signed informed consent.

### Preoperative management

The previous medical treatments were recorded before the operation, and physical examinations were performed. The Silfverskiöld test was performed to distinguish the contracture of the gastrocnemius-soleus complex from the Achilles tendon. The dorsal stretch < 10°, regardless of the stretch or flexion of the knee joint, indicated the contracture of the Achilles tendon. The dorsal stretch > 10° when flexing and < 10° when stretching the knee joint indicated the contracture of gastrocnemius-soleus complex. The hindfoot valgus, “too many toes” sign, and arch height were evaluated in the weight-bearing position. One foot and two feet heel-lifting test was performed for all patients to assess the capability of correcting the PTT deformity and strength.

### Operation method

Patients were placed in a supine position and given general plus nerve block anesthesia. A pneumatic tourniquet was applied to the proximal thigh, and the area was disinfection by entoiodine placing a surgical drape. The auxiliary surgery was performed according to the presence of contracture of gastrocnemius or Achilles tendon shown in preoperative physical examinations. For patients with Achilles tendon contracture, the percutaneous tri-semitendinostomy Achilles tendon lengthening was performed, and for patients with contracture of gastrocnemius, Strayer procedure was performed to release gastrocnemius aponeurosis.

Firstly, a transverse incision of approximately 1.5 cm was made at the tarsal sinus area anterior-inferior to the lateral malleolus. Blunt dissection of subcutaneous tissues, soft tissues, and ligaments was performed to expose the tarsal sinus and deep tissues. The hindfoot was passively introverted, the tarsal sinus was opened, and a guidewire was inserted. The appropriate size will allow the calcaneal subtalar joint complex to evert to approximately 2°–4°. Intraoperative imaging, to evaluate the degree of correction and placement of the trial implant, was performed to determine the correct position on the A/P view, so that the leading edge of the trial implant should approach, but not cross, the longitudinal bisection of the talus. The trailing edge of the implant should be at least 5 mm medial to the lateral wall of the calcaneus. The trial implant was then removed, and the proper sized implant was inserted. The appropriate size will allow 2°–4°of subtalar joint eversion. Intraoperative imaging was essential for this procedure, to verify proper positioning of the implant. Then, the provisional mode was removed, and the same type of HyProCure subtalar stabilizer was implanted.

**Fig. 1:**
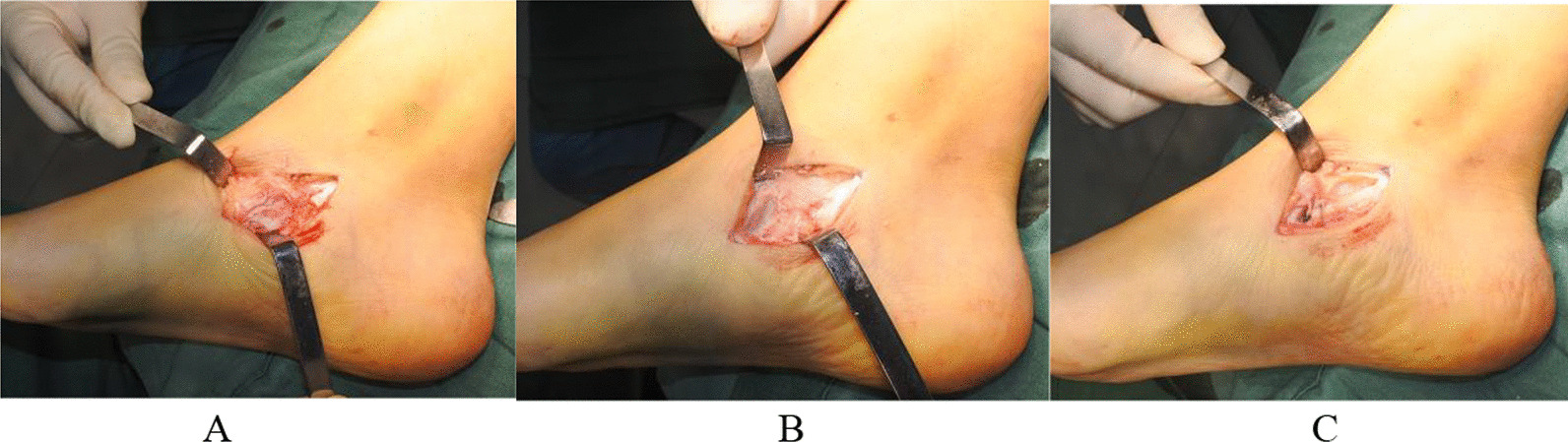
A 3–4 cm longitudinal incision was made along the medial foot to expose only the PTT insertion and accessory navicular (**A**). In addition to accessory navicular resection, the PTT insertion was cleared, and the medial surface of the navicular plantar was modified to a coarse surface to adjust the PTT tension (**B**). The sutured anchors were used to fix the PTT to the anterior-inferior site to the tubercle of the navicular bone (**C**). The arch was lifted, and the foot was placed supinely during suturing to restore the alignment direction of PTT and improve the longitudinal arch

Secondly, a longitudinal incision was made at the medial foot, starting from below the medial malleolus through the surface of the tubercle of the navicular bone and ending at the lower margin of the first metatarsal base. The length of the incision varied according to the surgical conditions. For patients with no loosening of medial deltoid ligament or spring ligament, only the PTT insertion and accessory navicular were exposed to treat the painful accessory navicular. For patients with type I accessory navicular, the accessory navicular was removed, and the PTT insertion was debrided. The medial surface of the plantar navicular was modified to a coarse surface, the PTT tension was adjusted, and sutured anchors were used to re-fix it to the anterior-inferior site on the tubercle of the navicular bone. The arch was lifted, and the foot was placed in a supine position during suturing to restore the alignment direction of PTT and improve the longitudinal arch. For patients with type II accessory navicular, the internal fixation method was selected according to the quality of the osteotomy surface of accessary navicular. If the bone fragment was relatively large and the bone mass at the osteotomy surface of accessary navicular was sufficient, two cannulated screws with a diameter of 2.5 mm were used to fix the osteotomy surface. If the bone mass at the osteotomy surface was insufficient, sutured anchors were used to reconstruct the PTT insertion to the plantar side and distal end of the navicular. For patients with type III accessory navicular, osteotomy and correction were performed, and the PTT insertion was reconstructed similar to that in patients with type II accessory navicular (Fig. 1). For patients with unstable medial ankle joints or with evident loosening of superficial deltoid ligament and spring ligament, sutured anchors were used to tighten the deltoid and spring ligaments. For a few patients with unstable medial soft tissues, flexor digitorum longus transfer and drilling of tubercle of navicular were performed, and the tendon was folded back and sutured to the proximal tendon to enhance the PTT function. Subsequently, the correction of deformity was re-examined, the location of internal fixation was confirmed by fluoroscopy, and the incision was irrigated and sutured layer-by-layer.

### Postoperative treatment and efficacy assessment

After the operation, aseptic dressing and elastic bandage were used for pressure dressing, and the ankle joint was immobilized by plaster at a neutral position with slight supination. The patients were recommended bed rest with the affected limb lifted, cryotherapy was performed to reduce swelling, and antibiotics were administered to prevent infection. The rehabilitation of the muscle groups around the ankle joint was started on day 2 after the operation, and the stitches were removed after 2 weeks. The patients could gradually achieve weight-bearing standing at 2–3 months postoperatively. After the arch recovered, the patients needed to readapt until the foot functions recovered completely. About 1 year after surgery, the patient basically adapted to normal movement.

Patients were followed up by specifically trained clinicians at outpatient departments. Additional follow-up was necessary for patients with any abnormalities. The function restoration and relevant complications were recorded. Clinical outcomes were assessed before the operation and at the last follow-up, according to the American Orthopedic foot and ankle society (AOFAS) ankle-hindfoot scale [14] (100-point system) and visual analog scale (VAS; 10-point system).

Radiological assessment: Talar first metatarsal angle (T1MT) and talonavicular coverage angle (TNCA) on anteroposterior foot view at the weight-bearing position, the Meary’s angle and calcaneal pitch angle on lateral foot X-ray images at the weight-bearing position, and calcaneus valgus angle (CVA) on hindfoot alignment view were assessed.

The satisfaction degree of the patients was assessed at the last follow-up, according to the pain, modification of shoes, activities in daily lives and exercises, and requirements of secondary surgery. The efficacy was classified as excellent, good, fair, and poor (Table 1) [6].

**Table 1:** System for assessing the satisfactory degree of patients

Grade	Status of patients
Excellent	No pain in the foot and no modifications of shoes
Good	No pain in the foot but with modifications of shoes
Fair	Mild pain in the foot during exercise; no mobility restriction but varying degrees of shoe modifications
Poor	Moderate pain in the foot, motility restriction, and varying degrees of shoe modifications

### Statistical analysis

SPSS 22.0 software (IBM Corp, Chicago, IL, USA) was used for statistical analysis. Qualitative data were described by frequencies (percentages), and quantitative data were represented as means and standard deviations. Paired *t *test was used for the comparison before and after the operation. *P* < 0.05 was considered statistically significant.

## Results

All patients achieved excellent wound healing, and no tarsal sinus implant loosening was detected. The operation duration was 48.34 ± 14.15 min. Complete follow-up data were acquired for 35 patients (50 feet), and one patient was lost to follow-up due to the change in contact information. The present cohort included 11 males and 24 females, and the disease was in bilateral feet in 15 patients (including six males and nine females), left foot in 23 patients, and right foot in 27 patients. The mean age of the patients was 11.56 ± 1.73 years, and the mean follow-up time of patients was 30 ± 9.3 months (Table 2). All the patients underwent accessory navicular debridement, PTT insertion reconstruction, and subtalar arthroereisis. Subsequently, 20 feet (40%) underwent percutaneous tri-semitendinostomy Achilles tendon lengthening, 25 feet (50%) underwent the release of gastrocnemius aponeurosis, and 10 feet (20%) underwent deltoid ligament or spring ligament tightening. Herein, 16, 28, and 6 feet exhibited type I, II, and III accessory navicular, respectively.

**Table 2:** General characteristics of patients

*n*	35 patients (50 feet)^b^
Male/female	17:33 (feet)
Left/right	23:27 (feet)
Age (year)	11.56 ± 1.73^a^
Mean follow-up time (months)	30 ± 9.3^a^
Operation time (min)	48.34 ± 14.15^a^
Intraoperative blood loss volume (mL)	21.92 ± 8.49^a^
Hospital stay (days)	7.02 ± 2.25^a^

^a^Data are described as means and standard deviations

^b^A total of 36 patients (51 feet) underwent this surgery. One patient with one foot involved was lost to follow-up. Finally, 35 patients (50 feet) were followed up

### Functional outcomes

The AOFAS score increased from 53.44 ± 5.03 points before the operation to 91.06 ± 3.97 points at the last follow-up, and the VAS score decreased from 5.56 ± 0.88 points before the operation to 0.60 ± 0.57 points at the last follow-up (*P* < 0.001; Table 3).

**Table 3:** Comparison of functional outcomes and imaging parameters before operation and at the last follow-up

Parameter	Before operation^a^	Last follow-up^a^	*P*
AOFAS scores	53.44 ± 5.03	91.06 ± 3.97	< 0.001
VAS scores	5.56 ± 0.88	0.60 ± 0.57	< 0.001
T1MT (°)	17.82 ± 6.12	3.80 ± 1.49	< 0.001
TNCA (°)	21.17 ± 5.39	3.83 ± 1.33	< 0.001
Meary’s angle (°)	17.83 ± 5.20	3.87 ± 2.04	< 0.001
Calcaneal pitch angle (°)	14.40 ± 3.75	17.84 ± 3.75	< 0.001
CVA (°)	14.15 ± 3.59	3.87 ± 1.55	< 0.001

*AOFAS* American Orthopedic Foot and Ankle Society ankle-hind- foot Score, *VAS* visual analog scale, *T1MT* Talar first metatarsal angle, *TNCA* talonavicular coverage angle, *CVA* calcaneus valgus angle

^a^Data are described by means and standard deviations

### Radiological outcomes

**Fig. 2:**
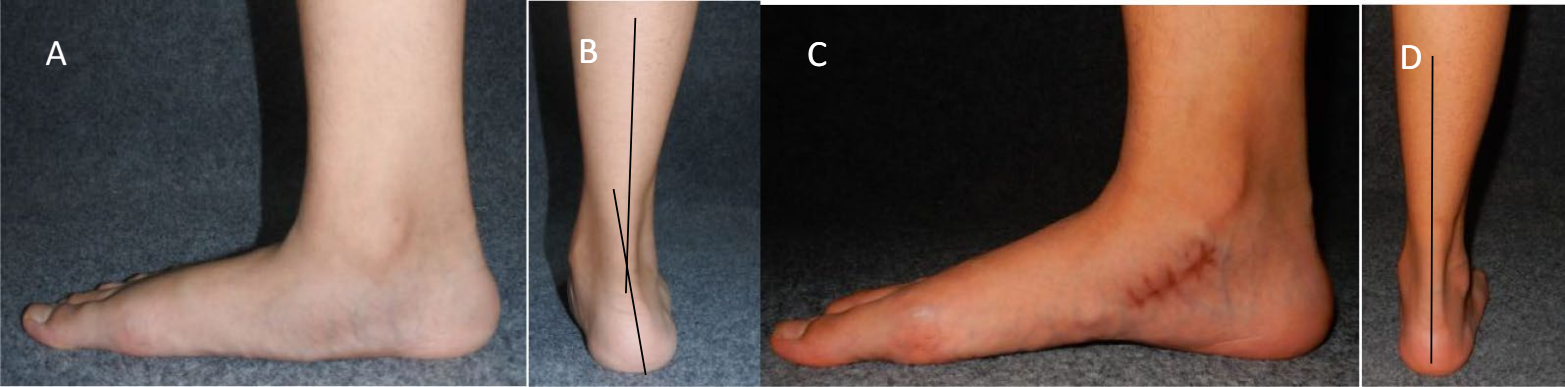
A 12-year-old boy of flexible flatfoot with accessory navicular in the right foot that underwent subtalar arthroereisis combined with medial soft tissue reconstruction. The patient showed a medial longitudinal arch collapse in the right foot (**A**) and evident hindfoot valgus deformity (**B**) before the operation. Compared to before the operation, the medial longitudinal arch appeared (**C**) and hindfoot alignment restored (**D**) at the last follow-up

**Fig. 3:**
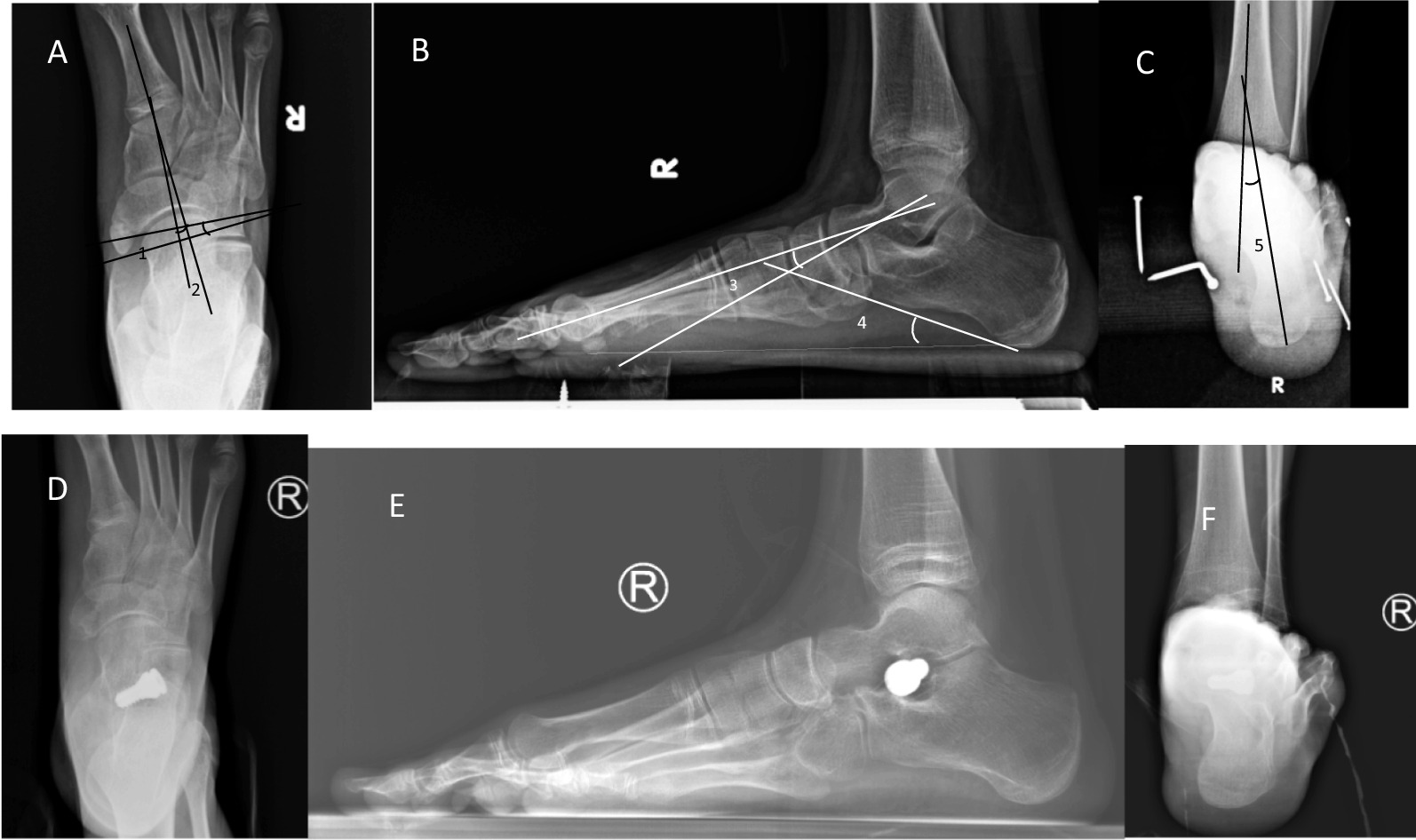
Anteroposterior and lateral X-ray images at weight-bearing position before the operation (**A**–**C**) T1MA of 6.1°, TNCA of 10.3°, Meary’s angle of 12.9°, and pitch angle of 17.1°. X-ray at Saltzman position before the operation showed evident valgus of right calcaneus, and CVA was 12°. Postoperative X-ray images showed that the arch was restored (**D**–**F**), T1MA was 4.4°, TNCA was 5.5°, Meary’s angle was 5.4°, and pitch angle was 20.3°. Preoperative X-ray images at Saltzman position show corrected deformity of the right foot, and the CVA was 3° (**F**). The numbers 1, 2, 3, 4, and 5 indicated TNCA and T1MA on the anteroposterior X-ray image at weight-bearing position, Meary’s angle, pitch angle on the lateral X-ray image at weight-bearing position, and calcaneus valgus on the X-ray image of Saltzman position. The normal range of the five imaging measurements: T1MA 0°–20°, average 7.7°, TNCA less than 7°, Meary’s angle ± 4°, pitch angle 20°–30° and CVA less than 5°

On the anteroposterior X-ray image at the weight-bearing position, the T1MT reduced from 17.82 ± 6.12° before the operation to 3.80 ± 1.49° at the last follow-up, and TCNA reduced from 21.17 ± 5.39° before the operation to 3.83 ± 1.33° at the last follow-up. On the lateral X-ray image at weight-bearing position, the Meary’s angle decreased from 17.83 ± 5.20° before the operation to 3.87 ± 2.04° at the last follow-up, and the pitch angle changed from 14.40 ± 3.75° before the operation to 17.84 ± 3.75° at the last follow-up. The CVA on hindfoot alignment image decreased from 14.15 ± 3.59° before the operation to 3.87 ± 1.55° at the last follow-up. The differences in all the five radiological parameters before and after the operation were statistically significant (*P* < 0.001; Table 3, Figs. 2, 3).

**Fig. 4:**
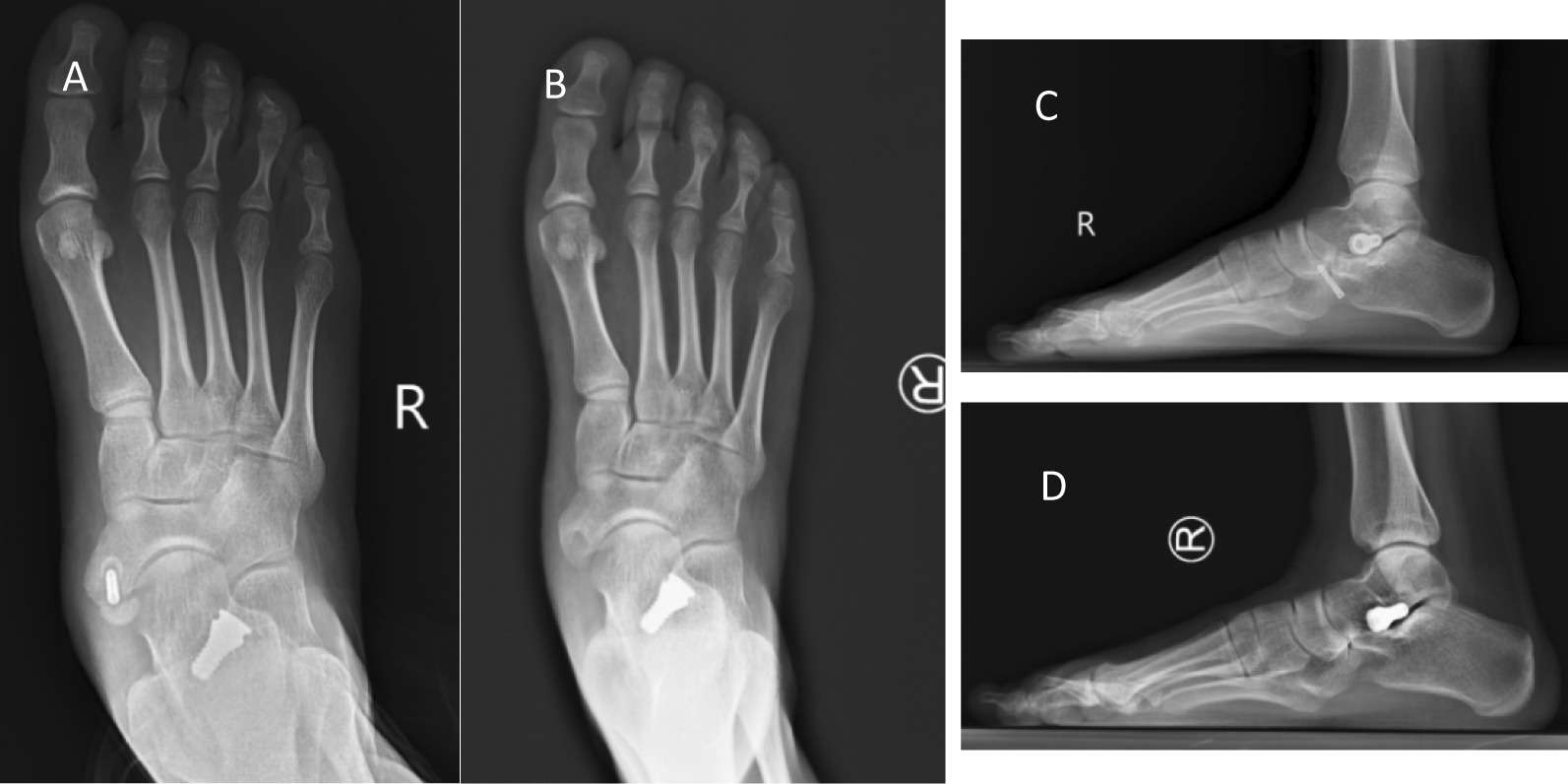
Screw was used for fixation in one patient. The 10-month follow-up X-ray showed that the accessory navicular was nonunion (**A**, **C**), and revision surgery was performed. The screw was removed, the accessory navicular was resected, and sutured anchor was used for reconstruction of PTT insertion. The functions and appearance improved substantially at the last follow-up, compared to the conditions before the operation (**B**, **D**). We speculated that when treating flatfoot with accessory navicular, complete resection of accessory navicular and using the sutured anchor to reconstruct PTT insertion is efficacious due to the poor blood flow for navicular

Complications occurred in three patients after the operation. One patient had pain in the tarsal sinus area at 3 months after the operation, and the pain was alleviated after blocking of the tarsal sinus and symptomatic treatment by NSAIDs. One patient exhibited pain and swelling in the tarsal sinus area after the operation, which worsened after walking. X-ray images and physical examinations showed that the screw is extremely large. After the size of the tarsal sinus screw was adjusted, the postoperative pain was alleviated. One patient presented nonunion of screw fixation of accessory navicular by X-ray imaging, which was treated by a sutured anchor for the reconstruction of PTT insertion. The wound in the patient healed at the last follow-up (Fig. 4).

## Discussion

Accessory navicular is the most common tarsal deformity, occurring primarily in females. The majority of patients with accessory navicular were asymptomatic, and some may have several symptoms, including pain, pressing pain, and swelling in the medial accessory navicular areas, which could be worsened during weight-bearing, walking, movement, and wearing shoes. The pressure on the bone, abnormal biomechanics, and repeated injuries on the cartilage of fibrocartilaginous joint could induce pain, mainly diagnosed by clinical and radiological features. X-ray combined with magnetic resonance imaging (MRI) diagnosed the presence of accessory navicular and synchondrosis. The initial treatment for accessory navicular is conservative treatment, such as fixation or using orthotics. However, occasionally, such treatments do not alleviate symptoms. The surgical treatment is successful in most cases in alleviating pain [4, 10, 15, 16].

Accessory navicular resection combined with PTT insertion reconstruction alleviated pain in patients and restored the PTT functions, as well as partially restored the arch, while the alterations of foot biomechanics are extremely low [4, 17]. Previous studies have shown that irrespective of Kidner procedure or fusion of accessory navicular increased the calcaneal pitch angle but did not significantly influence the TNCA and T1MT, according to radiological findings [7, 18]. Patients with persistent pain after simple accessory navicular resection worsened the flatfoot deformity, in which radiological records show the gradual decrease in longitudinal arch [19]. Although the causes of recurrent pain after Kidner procedure are yet unclear, these could be associated with hindfoot eversion and/or foot deformity, which increases the tension at the PTT grafting site, consequently leading to pain and PTT dysfunction [7]. The findings in this study showed that the radiological parameters improved, and the postoperative appearance improved substantially. Therefore, resecting accessory navicular is not a definite treatment for flexible flatfoot patients with painful accessory navicular [1, 3, 5–7].

Graham et al. [20] proposed the conception of subtalar stabilization and suggested that the morphology of HyProCure screws matches the anatomical structure of the tarsal sinus. Biomechanical experiments demonstrated that HyProCure screws reduce the tendency of subluxation of talus from calcaneus, thereby evenly distributing the axial stress anterior and posterior to tarsal sinus, restoring the normal motion axis of subtalar joint, correcting the hindfoot alignment, restoring the arch, and eliminating the over-eversion and significantly reducing the mechanical load on PTT insertion. Therefore, the HyProCure screws alleviated pain and corrected the flatfoot deformity [21, 22]. Ruiz-Picazo et al. [23] suggested that subtalar arthroereisis is an effective method for the treatment of flexible flatfoot, with good postoperative functions and radiological outcomes. However, the efficacy of subtalar arthroereisis for flatfoot patients with accessory navicular is yet unclear. In a prospective study consisting of 20 patients, Garras et al. [1] demonstrated that modified Kidner procedure combined with subtalar stabilization alleviates pain in accessory navicular area and corrects flatfoot deformity. The correction of deformity could be maintained even after the graft was removed, which could be associated with the restoration of appropriate length and tension after the PTT recovered at the site of reconstruction, supporting the arch when the prosthesis was removed. Therefore, for flatfoot patients with accessory navicular, the resection of accessory navicular combined with correct identification of flatfoot deformity achieved better clinical outcomes [24].

The complications of subtalar arthroereisis include persistent pain in the talar sinus area, movement disorders, ischemic necrosis of the talus, cyst in the talus, hardware dislocation, foreign-body reactions, and subtalar arthritis [1, 23]. Postoperative pain in the talar sinus area is the most common complication, and the implants need to be removed from as high as one third of patients. Such pain could be associated with the implantation of inappropriate sizes or over-correction in procedures [25]. In this study, one patient presented swelling and pain in the talar sinus area, which worsened after walking. X-ray and physical examinations showed that the screw was extremely large. After the size of the talar sinus screw was adjusted, the pain in the postoperative follow-up alleviated. For another patient, the pain was alleviated by blockade in the talar sinus area and symptomatic treatment by NSAIDs. Foreign-body reactions to grafts and subtalar arthritis have been rarely reported [25].

Some studies have reported PTT dysfunctions in pediatric patients, and the most relevant cases exhibit traumatic PTT rupture. The current clinical practices showed that most flatfoot patients have substantial loosening of medial soft tissues, which is persistent even after subtalar arthroereisis. Therefore, auxiliary medial soft tissue-enhancing procedures were performed in our practice, and the immediate stabilization effect was evident in operation and the function was restored satisfactorily.

In this study, 35 pediatric flexible flatfoot (50 feet) with painful accessory navicular were treated with subtalar arthroereisis combined with medial soft tissue reconstruction, which improved the clinical and radiological manifestations. The patients recovered rapidly after the operation with only a very few complications. Especially, the postoperative movement capability of patients improved significantly after the operation with high satisfaction degrees of the patients and their families. This treatment fulfills the requirements of the minimal surgical wound, rapid postoperative recovery, and short non-weight-bearing time while alleviating the pain in patients.

Nevertheless, the present study has some limitations. This was a retrospective study, and patients were followed up for a short period. Thus, there is no study on whether the removal of implants will affect the efficacy, data from longer follow-up are required.

In summary, the procedures of subtalar arthroereisis combined with medial soft tissue reconstruction are simple and minimally invasive, and the short-term follow-up findings were satisfactory. This treatment alleviated the pain and improved the functions in pediatric patients with painful accessory navicular accompanied by flexible flatfoot.

## Data Availability

The data and materials used and/or analyzed during the current study are available from the corresponding author on reasonable request.
